# The Rising Inpatient Burden of Metabolic Dysfunction-Associated Steatotic Liver Disease: Insights From the Nationwide Inpatient Sample (2018-2022)

**DOI:** 10.7759/cureus.105858

**Published:** 2026-03-25

**Authors:** Muhammad Haris Latif, Imran Khokhar, Ayesha Kang, Eman Mazhar, Ali Haider, Kahee A Mohammed, Hani El-Halawany, Kamran Qureshi

**Affiliations:** 1 Internal Medicine, SSM Health St. Mary's Hospital, Saint louis, USA; 2 Internal Medicine, Reading Tower Health, Reading, USA; 3 General Surgery, Bronx Lebanon Hospital, New York, USA; 4 Internal Medicine, SSM Health St. Mary's Hospital, Saint Louis, USA; 5 Internal Medicine, Nishtar Medical University, Multan, PAK; 6 Gastroenterology, SSM Health Depaul Hospital, Saint Louis, USA; 7 Hepatology, Saint Louis University School of Medicine, Saint Louis, USA

**Keywords:** comorbid metabolic disease, healthcare resource utilization, hospitalized patients, in-hospital mortality, inpatient outcomes, length of stay, metabolic dysfunction–associated steatotic liver disease (masld), nationwide inpatient sample (nis), prevalence, temporal trends

## Abstract

Background and objective

Metabolic dysfunction-associated steatotic liver disease (MASLD) is increasingly common and is a growing public health concern. However, data on MASLD prevalence and outcomes among hospitalized adults in the United States are limited. Therefore, this study sought to evaluate the inpatient burden of MASLD, along with associated outcomes and patterns of resource utilization.

Methods

We analyzed 2018-2022 Nationwide Inpatient Sample (NIS) data for adult MASLD hospitalizations using the International Classification of Diseases, 10th Revision (ICD-10) codes and excluded secondary liver diseases. Survey-weighted multivariable models assessed the prevalence of the condition and the association between MASLD and inpatient mortality, adjusting for demographic, clinical, and hospital-level confounders. Sensitivity models included cirrhosis and hepatocellular carcinoma (HCC).

Results

Among 144 million adult hospitalizations, the prevalence of MASLD rose from 1.77% to 3.90% during the surveyed period. Crude MASLD mortality was lower in 2018-2021 but higher in 2022. Adjusted models showed year-to-year variation in the association with mortality. Including cirrhosis and HCC in the model showed that MASLD was associated with lower mortality, but the results depended on model adjustments. Older age, cardiovascular disease, chronic kidney disease, cancer, and cirrhosis were predictors of mortality among patients with MASLD.

Conclusions

The rate of MASLD among hospitalized adults in the United States rose from 2018 to 2022. Associations with mortality differed across years and depending on the analytic adjustments used. These findings at the discharge level should not be taken as evidence of either beneficial or harmful effects of MASLD.

## Introduction

Metabolic dysfunction-associated steatotic liver disease (MASLD), formerly known as nonalcoholic fatty liver disease (NAFLD), is the most common chronic liver disorder worldwide [1]. MASLD represents a reclassification of NAFLD based on positive metabolic criteria; however, administrative datasets lack a dedicated International Classification of Diseases, Tenth Revision, Clinical Modification (ICD-10-CM) code for MASLD, and legacy NAFLD coding may incompletely capture cases. It affects individuals with hepatic steatosis and at least one cardiometabolic risk factor, such as obesity, diabetes, dyslipidemia, or hypertension, while excluding other causes [2]. MASLD affects about one-third of adults globally and in the United States, with projections that may reach approximately 33.5% by 2030, representing a substantial and increasing public health burden [3].

While the epidemiology of MASLD is well described in the general population, with a global prevalence of 25.24%, recent data on its prevalence and outcomes among hospitalized patients remain scarce. MASLD is frequently underdiagnosed in high-risk groups, particularly individuals with diabetes, highlighting the importance of early detection. Previous inpatient studies have provided valuable insights; however, most were conducted before the adoption of MASLD definitions [4], were limited to earlier time periods, or focused on specific subpopulations or outcomes. National analyses have demonstrated increasing hospitalizations for NAFLD and disparities by age, sex, and race, but contemporary longitudinal data aligned with current MASLD definitions are lacking.

The primary objectives of this study were to quantify temporal trends in the coded inpatient prevalence of MASLD among adult U.S. hospitalizations from 2018 to 2022 and to evaluate the association between MASLD coding and in-hospital mortality using year-specific discharge-level analyses. We additionally examined predictors of inpatient mortality among hospitalizations with MASLD.

## Materials and methods

Data source

We conducted a retrospective, observational cohort study using data from the Healthcare Cost and Utilization Project (HCUP) National Inpatient Sample (NIS) from 2018 through 2022. The NIS is the largest publicly available all-payer inpatient database in the United States, capturing a 20% stratified sample of discharges from non-federal acute care hospitals across participating states [5]. The database has been widely used to estimate national disease prevalence, healthcare utilization, and inpatient outcomes. Each hospitalization is de-identified and treated as a unique entry. Institutional review board approval was not required because the NIS contains publicly available, de-identified data.

Study population

The study population included all adult hospitalizations (aged ≥ 18 years) captured in the NIS during the study period. ICD-10-CM diagnosis codes were used to identify eligible hospitalizations and associated clinical conditions.

Inclusion and exclusion criteria

Hospitalizations were stratified into two groups based on the presence or absence of MASLD. As there is no unique ICD-10-CM code for MASLD, hospitalizations were classified as MASLD if ICD-10-CM diagnosis codes indicating NAFLD, hepatic steatosis, or steatohepatitis were present, including codes for fatty change of the liver, steatohepatitis, nonalcoholic fatty liver disease, or metabolic-associated steatohepatitis [2]. To improve diagnostic specificity and align with contemporary MASLD consensus definitions, hospitalizations were additionally required to have at least one documented metabolic risk factor, including obesity (E66.xx), type 2 diabetes (E11.xx), hypertension (I10-I15), or dyslipidemia (E78.xx). Because obesity, diabetes, hypertension, and dyslipidemia were components of the MASLD identification algorithm, these variables were excluded from the primary MASLD-versus-non-MASLD mortality model to avoid overadjustment.

Hospitalizations among patients younger than 18 years and those with missing demographic or in-hospital mortality data were excluded. Hospitalizations with alternative causes of chronic liver disease were also excluded, including alcohol-associated liver disease or alcohol use disorder, chronic viral hepatitis (hepatitis B or C), autoimmune hepatitis, hemochromatosis, Wilson disease, and other secondary liver diseases. Similar ICD-10-CM-based approaches have been used in prior large administrative database studies to identify MASLD [5].

Statement on misclassification

Due to the inpatient and administrative nature of the NIS, the specific diagnostic modality used to evaluate hepatic steatosis (such as imaging, histology, or laboratory testing) could not be ascertained, and cases may have been underrecognized or misclassified [6]. The final analytic cohort comprised approximately 144 million weighted adult hospitalizations nationwide. Conducting an NAFLD-only sensitivity analysis would further improve reproducibility. As the current revision cycle utilized a finalized analytic dataset, the coding algorithm has been clarified in detail, and this narrower sensitivity analysis is highlighted as an important direction for future research.

Study variables

We collected patient-level demographic information, including age (categorized as 18-44 years, 45-64 years, and ≥65 years), sex, race/ethnicity, primary insurance payer, and median household income quartile by ZIP code. Hospital characteristics collected included geographic region, hospital bed size, teaching status, and urban versus rural location, as defined by HCUP. We also identified major clinical comorbidities relevant to MASLD and inpatient mortality, including coronary artery disease, congestive heart failure, chronic kidney disease, chronic obstructive pulmonary disease, cirrhosis, hepatocellular carcinoma (HCC), hepatitis C virus (HCV) infection, alcohol-related liver disease, and other liver diseases.

Outcome measures

The primary outcome was the annual prevalence of MASLD among hospitalized adults in the United States from 2018 to 2022. Secondary outcomes included in-hospital mortality, length of stay (LOS), and total hospital charges. LOS and total hospital charges were further analyzed using year-specific survey-weighted linear regression with log-transformed outcomes, with adjusted percent differences calculated as 100 × (exp(beta) − 1) [7]. We also evaluated factors predicting in-hospital mortality among hospitalizations with MASLD.

Statistical analysis

All analyses accounted for the complex NIS survey design using discharge weights (DISCWT), hospital clustering (HOSP_NIS), and stratification (NIS_STRATUM). Weighted annual prevalence estimates were calculated for each study year. Baseline characteristics were compared using survey-weighted mean estimates for continuous variables and survey-weighted cross-tabulations for categorical variables. Crude inpatient mortality was estimated according to MASLD status for each year.

Year-specific survey-weighted multivariable logistic regression models were then used to evaluate the association between MASLD and in-hospital mortality. The primary model adjusted for year-specific demographic characteristics, hospital characteristics, and major non-metabolic comorbidities and competing liver disease etiologies, including congestive heart failure, coronary artery disease, stroke, chronic kidney disease, chronic obstructive pulmonary disease, cancer, HCV infection, alcohol-related liver disease, and other liver disease. Cirrhosis and HCC were not included in the primary model because they may represent downstream markers of disease severity. A secondary sensitivity model additionally included cirrhosis and HCC. Separate MASLD multivariable models were used to identify predictors of inpatient mortality among hospitalizations meeting the stricter MASLD definition. Adjusted odds ratios (aORs) with 95% confidence intervals (CIs) were reported, and a p-value < 0.05 was considered statistically significant.

## Results

Study population

**Figure 1:**
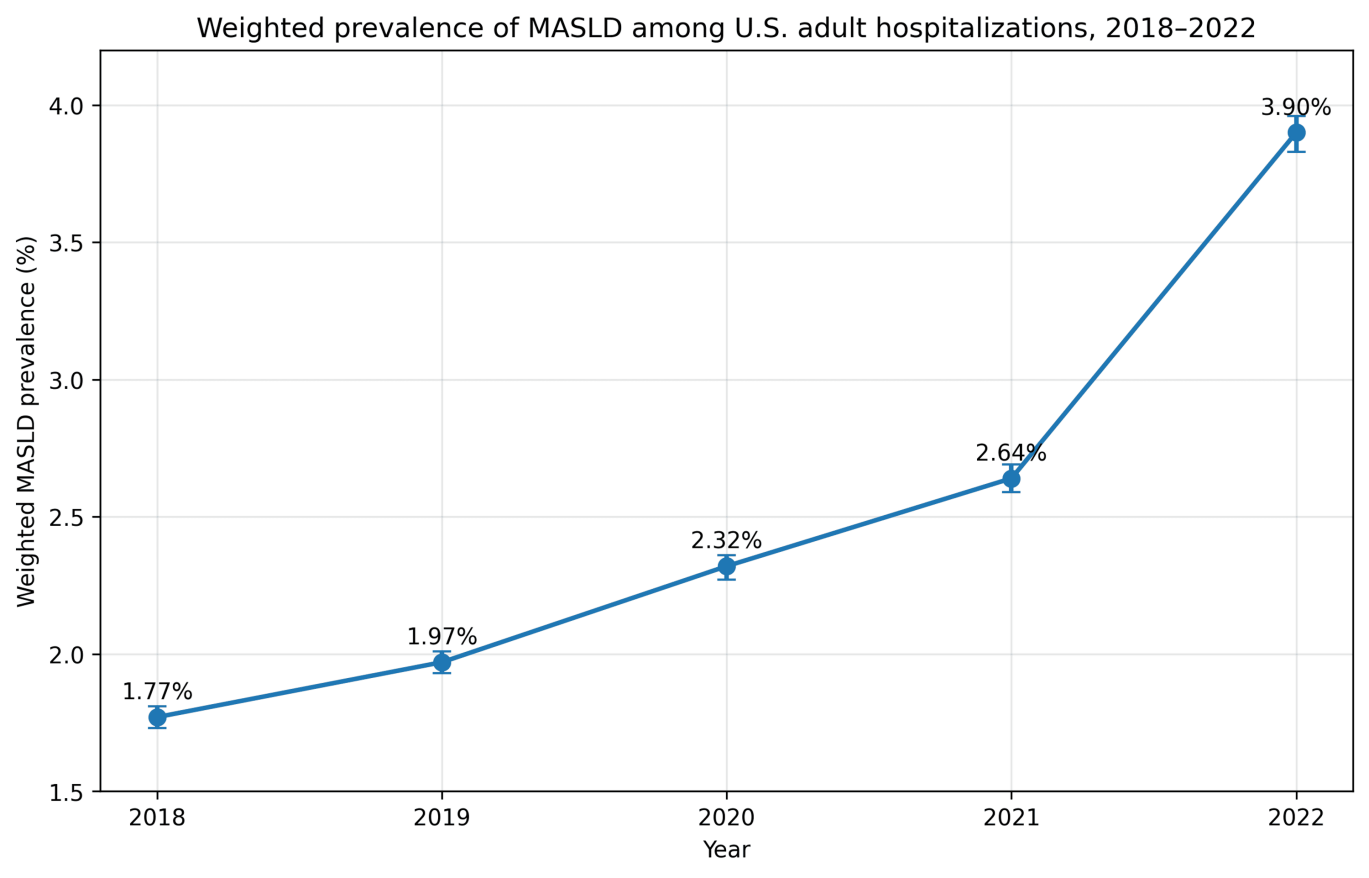
Temporal trends in MASLD prevalence (2018–2022) The figure depicts the annual weighted prevalence of MASLD among adult hospitalizations in the United States. Prevalence estimates were generated using survey-weighted analyses of the National Inpatient Sample. The increasing trend over time was statistically significant (p for trend <0.001) MASLD: metabolic dysfunction-associated steatotic liver disease

From 2018 to 2022, the NIS included approximately 144 million weighted adult hospitalizations. The coded inpatient prevalence of MASLD increased steadily over time, from 1.77% (95% CI: 1.73%-1.81%) in 2018 to 1.97% (95% CI: 1.93%-2.01%) in 2019, 2.32% (95% CI: 2.27%-2.36%) in 2020, 2.64% (95% CI: 2.59%-2.69%) in 2021, and 3.90% (95% CI: 3.83%-3.96%) in 2022. (Table 1, Figure 1).

**Table 1:** Weighted inpatient prevalence of MASLD by year MASLD: metabolic dysfunction-associated steatotic liver disease; CI: confidence interval

Year	Sampled discharges, n	Weighted national admissions, n	Weighted MASLD prevalence, % (95% CI)
2018	60,52,246	3,02,61,223	1.77 (1.73–1.81)
2019	60,43,912	3,02,19,558	1.97 (1.93–2.01)
2020	55,33,733	2,76,68,666	2.32 (2.27–2.36)
2021	56,88,609	2,84,43,009	2.64 (2.59–2.69)
2022	55,72,882	2,78,64,400	3.90 (3.83–3.96)

Baseline characteristics of MASLD hospitalizations

Across 2018-2021, hospitalizations with MASLD were slightly younger and less often female than non-MASLD hospitalizations, whereas in 2022, MASLD admissions were modestly older. Across all years, MASLD admissions were more frequently Hispanic, more commonly treated at urban teaching hospitals, and more often admitted to large hospitals.

Healthcare resource utilization

In year-specific survey-weighted multivariable linear regression models using log-transformed outcomes, MASLD admissions had 8.7% to 9.7% longer LOS and 19.8% to 24.2% higher total hospital charges than non-MASLD admissions after adjusting for demographic characteristics, hospital factors, major comorbidities, and competing liver disease etiologies (see Appendices).

In-hospital outcomes

Crude inpatient mortality was lower among MASLD admissions from 2018 through 2021, but higher in 2022. In the primary year-specific multivariable models, which excluded cirrhosis and HCC, MASLD was not independently associated with inpatient mortality in 2018 (aOR: 1.01, 95% CI: 0.96-1.06), 2019 (aOR: 1.03, 95% CI: 0.99-1.08), or 2021 (aOR: 0.99, 95% CI: 0.96-1.03), was associated with modestly lower mortality in 2020 (aOR: 0.93, 95% CI: 0.89-0.96), and higher mortality in 2022 (aOR: 1.33, 95% CI: 1.29-1.37).

In sensitivity analyses that additionally included adjustments for cirrhosis and HCC, MASLD appeared to be associated with lower inpatient mortality across all years. Because cirrhosis and HCC likely represent downstream markers of liver disease severity, these sensitivity models were interpreted cautiously as possibly overadjusted rather than as evidence of a protective clinical effect, as presented in Table 2.

**Table 2:** Year-specific multivariable association between MASLD and inpatient mortality (2018–2022) and hospital characteristics of hospitalizations with MASLD Primary model adjusted for age, sex, race, payer, ZIP-income quartile, hospital region, teaching/location status, bed size, CHF, CAD, stroke, CKD, COPD, cancer, HCV infection, alcohol-related liver disease, and other liver disease. The sensitivity model additionally included cirrhosis and HCC MASLD: metabolic dysfunction-associated steatotic liver disease; aOR: adjusted odds ratio; CI: confidence interval; CHF: congestive heart failure; CAD: coronary artery disease; COPD: chronic obstructive pulmonary disease; HCV: hepatitis C virus; HCC: hepatocellular carcinoma

Year	Primary model, aOR (95% CI)	P-value	Sensitivity model, aOR (95% CI)	P-value
2018	1.01 (0.96–1.06)	0.735	0.80 (0.76–0.84)	< 0.001
2019	1.03 (0.99–1.08)	0.16	0.82 (0.79–0.86)	< 0.001
2020	0.93 (0.89–0.96)	< 0.001	0.77 (0.74–0.81)	< 0.001
2021	0.99 (0.96–1.03)	0.589	0.84 (0.81–0.87)	< 0.001
2022	1.33 (1.29–1.37)	< 0.001	0.74 (0.71–0.77)	< 0.001

Predictors of mortality across the cohort

Among hospitalizations meeting the MASLD definition, older age, male sex, cardiovascular comorbidity, chronic kidney disease, cancer, and cirrhosis were the most consistent predictors of inpatient mortality across years. Specifically, age, congestive heart failure, coronary artery disease, stroke, chronic kidney disease, cancer, and cirrhosis showed reproducible associations with higher inpatient mortality in most year-specific models (Table 3). Female sex was consistently associated with lower mortality. Metabolic variables such as hypertension, dyslipidemia, and obesity frequently showed inverse associations within MASLD admissions. Still, these findings were interpreted cautiously, given the likelihood of coding intensity effects, residual confounding, and case-mix differences inherent to administrative inpatient data [8,9].

**Table 3:** Year-specific survey-weighted multivariable logistic regression models of in-hospital mortality (2018–2022) Adjusted odds ratios were derived from survey-weighted multivariable logistic regression models. Models were adjusted for age, sex, race/ethnicity, insurance status, hospital characteristics, comorbidity burden, and relevant clinical comorbidities aOR: adjusted odds ratio; CHF: congestive heart failure; CAD: coronary artery disease; CKD: chronic kidney disease

Predictor	2018 aOR	2019 aOR	2020 aOR	2021 aOR	2022 aOR
Age (per year)	1.03	1.03	1.04	1.03	1.03
Female sex	0.8	0.8	0.85	0.78	0.9
CHF	1.79	1.76	1.79	1.36	1.61
CAD	1.26	1.38	1.25	1.23	1.24
Stroke	2.04	2.03	1.62	1.71	1.69
CKD	1.52	1.61	1.46	1.26	1.36
Cancer	2.43	2.29	1.61	1.37	1.79
Cirrhosis	2.02	1.76	1.37	1.35	1.88

## Discussion

In this nationally representative discharge-level analysis of U.S. hospitalizations from 2018 to 2022, the coded inpatient prevalence of MASLD increased steadily, rising from 1.77% in 2018 to 3.90% in 2022. This trend supports a growing inpatient burden of MASLD in the United States, even though the observed prevalence remains well below population-based estimates, likely reflecting underrecognition and undercoding within acute care settings [1]. Prior analyses of the NIS have reported a low but increasing prevalence of NAFLD among hospitalized patients, typically below 1% in earlier studies and approaching 1-2% in more recent ICD-10-era analyses. These values remain substantially lower than population-based prevalence estimates [4,5].

MASLD was identified through a reproducible ICD-10-based algorithm that incorporates metabolic risk factors and excludes alternative causes of liver disease, consistent with current consensus definitions. Survey-weighted analyses and multivariable adjustment for demographic, clinical, and hospital-level factors enhance the validity of national inferences. Collectively, these methodological features enable a comprehensive evaluation of inpatient MASLD burden, outcomes, and healthcare utilization, while also highlighting the persistent underrecognition of MASLD in hospitalized populations.

The association between MASLD and inpatient mortality was unstable across years and highly sensitive to model specification. In the primary models excluding cirrhosis and HCC, MASLD was neutral in 2018, 2019, and 2021; modestly inversely associated with mortality in 2020; and positively associated with mortality in 2022. In contrast, when cirrhosis and HCC were added in the sensitivity analysis, MASLD appeared to be associated with lower mortality in every year. Because cirrhosis and HCC likely lie on the pathway of liver disease progression, this latter result is best interpreted as evidence of overadjustment for downstream disease severity rather than a true protective effect of MASLD.

Beyond mortality, MASLD was consistently associated with greater inpatient resource utilization across all study years. The observed increases in LOS and total hospital charges suggest that MASLD contributes meaningfully to inpatient complexity and healthcare burden, even when mortality associations vary across years. Prior extensive longitudinal literature findings demonstrate that steatotic liver disease is associated with increased long-term risks of cardiovascular mortality, malignancy, and infection-related complications [1,4,10]. However, the present findings should be interpreted cautiously, given the inherent limitations of administrative data.

Among MASLD admissions, inpatient mortality was more consistently associated with older age, cardiovascular comorbidity, chronic kidney disease, cancer, and cirrhosis than with any single metabolic feature. These findings suggest that short-term inpatient mortality in MASLD reflects overall systemic illness burden and advanced liver disease rather than the presence of coded steatosis alone. These risk factors consistently predominate in inpatient mortality models across NIS studies. Previous NIS research on sepsis has shown poorer outcomes when steatotic liver disease coexists with severe infection [11,12]. This is especially true in patients with advanced phenotypes such as nonalcoholic steatohepatitis (NASH) or cirrhosis. This finding underscores the prognostic significance of infection and end-organ dysfunction in this population.

In contrast, female sex and certain cardiometabolic conditions - including dyslipidemia, hypertension, and obesity - were associated with lower adjusted odds of inpatient mortality [9]. These inverse associations, observed in large inpatient administrative datasets, are typically attributed to confounding by baseline risk and differences in illness severity at presentation. Variations in treatment exposure, such as increased use of guideline-directed cardiometabolic therapies, also play a role. These effects are seen as explanations, rather than inherent biological protection.

Several factors may explain this apparent discrepancy. First, MASLD as identified using ICD-10-CM coding may preferentially capture patients with recognized but less advanced disease, as the NIS lacks information on fibrosis stage or histologic severity, which are key prognostic determinants [5]. Second, undercoding of MASLD in critically ill patients is possible, particularly when acute life-threatening conditions take precedence in clinical documentation and billing priorities. Third, competing high-risk diagnoses such as sepsis, advanced heart failure, metastatic malignancy, or decompensated cirrhosis may overshadow chronic liver disease in discharge coding, thereby reducing the likelihood that MASLD is recorded in patients with the highest short-term mortality risk [5]. Collectively, these documentation patterns, case-mix differences, and the lack of disease-severity data may contribute to the observed association rather than indicating reduced biological risk.

Importantly, these inpatient findings should not be interpreted as evidence of reduced clinical severity or lower long-term risk in MASLD. Large longitudinal studies continue to show that steatotic liver disease is associated with substantial long-term cardiometabolic, oncologic, liver, and sepsis-related risk [13]. The present analysis instead reflects discharge-level associations within an administrative inpatient dataset, where coding practices, case mix, and the absence of fibrosis staging strongly influence observed mortality relationships.

In summary, the NIS, the largest all-payer inpatient database in the United States, was utilized to generate nationally representative estimates of MASLD prevalence and outcomes across diverse inpatient settings. The substantial sample size, exceeding 144 million weighted hospitalizations, allows for a rich assessment of temporal trends and relatively uncommon outcomes, such as in-hospital mortality. The analysis covers the contemporary ICD-10 era (2018 to 2022), thereby addressing limitations inherent in previous NAFLD-era inpatient studies.

Limitations

Several limitations are inherent to the retrospective, administrative design of this study. Firstly, identification of MASLD was based on ICD-10-CM diagnosis codes and associated metabolic risk factors, which may introduce misclassification and likely lead to an underestimation of the actual disease prevalence due to underrecognition and undercoding, especially during acute hospitalizations. Second, the NIS is a discharge-level database that does not permit longitudinal follow-up, thereby limiting the assessment of long-term outcomes or repeated hospitalizations at the individual patient level. Third, the NIS lacks detailed clinical information, including laboratory values, imaging findings, histology, medication use, and fibrosis severity. This absence precludes the evaluation of disease stage or differentiation between simple steatosis and more advanced MASLD phenotypes. Fourth, residual confounding may persist despite adjustment for a broad range of comorbidities and hospital-level factors because unmeasured variables such as outpatient care patterns and socioeconomic determinants are not fully captured. Finally, as the analysis focuses exclusively on hospitalized patients, the findings may not be generalizable to community-based or outpatient MASLD populations. Consistent with all observational studies, the associations observed should not be interpreted as evidence of causality.

## Conclusions

Based on our findings, the coded inpatient prevalence of MASLD rose markedly in the United States from 2018 to 2022. However, the association between MASLD and inpatient mortality was inconsistent across years and highly sensitive to the adjustment approach, particularly the inclusion of downstream liver disease severity variables such as cirrhosis and HCC. Hospitalization may represent an important opportunity to identify underlying metabolic liver disease and initiate outpatient risk stratification and preventive management. However, these discharge-level findings should not be interpreted as evidence of a protective or uniform mortality effect of MASLD.

## Appendices

**Table 4:** Supplementary table S1 - ICD-10-CM codes used for case identification, comorbidities, and exclusion criteria ICD-10-CM codes were identified from all available diagnosis fields in the NIS. MASLD was defined by the presence of a steatotic liver disease code plus ≥ 1 metabolic risk factor, with exclusion of alternative causes of chronic liver disease. Coding strategies are consistent with prior large administrative database studies and current MASLD consensus definitions. ICD-10-CM: International Classification of Diseases, Tenth Revision, Clinical Modification; NIS: Nationwide Inpatient Sample; MASLD: metabolic dysfunction–associated steatotic liver disease

Condition	ICD-10-CM codes
Fatty change of the liver	K76.0
Nonalcoholic fatty liver disease (NAFLD)	K76.0
Nonalcoholic steatohepatitis (NASH)	K75.81
Metabolic dysfunction-associated steatohepatitis (MASH)	K75.81
Obesity	E66.xx
Morbid obesity	E66.01
Type 2 diabetes mellitus	E11.xx
Hypertension	I10–I15
Dyslipidemia/hyperlipidemia	E78.xx
Coronary artery disease	I25.xx
Congestive heart failure	I50.xx
Chronic kidney disease	N18.xx
Chronic obstructive pulmonary disease	J44.xx
Cirrhosis	K74.60, K74.69
Hepatocellular carcinoma	C22.0
Sepsis	A40.xx, A41.xx
Septic shock	R65.21

**Table 5:** Supplementary table S2 - survey-weighted baseline characteristics of hospitalizations with and without MASLD (2018–2022) Values are survey-weighted percentages unless otherwise indicated. Age is presented as a survey-weighted mean. All year-specific comparisons between MASLD and non-MASLD groups were statistically significant for age, sex, race, primary payer, ZIP-income quartile, hospital region, hospital bed size, and hospital teaching/location status MASLD: metabolic dysfunction-associated steatotic liver disease

Characteristic	2018 non-MASLD	2018 MASLD	2019 non-MASLD	2019 MASLD	2020 non-MASLD	2020 MASLD	2021 non-MASLD	2021 MASLD	2022 non-MASLD	2022 MASLD
Mean age, years	58.2	57.1	58.43	57.24	58	56.58	58.05	56.43	58.61	59.63
Female, %	57.42	54.44	57.1	54.3	56.25	52.26	56.2	52.51	56.77	50.82
Race, %										
White	66.91	68.29	67.23	67.98	65.64	66.26	65.41	65.82	65.1	64.98
Black	15.19	9.96	15.42	10.06	15.96	10.84	15.78	10.85	15.65	11.01
Hispanic	11.44	15.22	10.96	15.44	11.78	16.18	12.28	16.96	12.53	17.39
Asian/Pacific Islander	2.79	2.48	2.8	2.51	2.84	2.54	2.9	2.57	3.01	2.84
Native American	0.64	0.89	0.68	0.95	0.72	0.99	0.71	0.93	0.69	0.94
Other	3.03	3.17	2.9	3.07	3.06	3.19	2.92	2.87	3.02	2.84
Primary payer, %										
Medicare	48.15	41.83	48.22	41.7	46.63	39.38	46.22	38.58	47.53	46.24
Medicaid	18.3	17.91	18.06	18.29	18.98	19.68	19.24	20.6	19.32	19.76
Private insurance	26.35	31.41	26.22	30.74	26.65	30.87	26.92	31.29	25.93	25.9
Self-pay	4.08	5.71	4.23	5.93	4.21	6.3	4.03	5.84	3.75	4.67
No charge	0.36	0.53	0.34	0.57	0.35	0.64	0.31	0.6	0.27	0.41
Other	2.77	2.61	2.92	2.77	3.17	3.13	3.28	3.09	3.19	3.02
ZIP-income quartile, %										
1st quartile	29.69	28.61	30.64	29.3	30.68	29.43	30.5	29.42	29.77	30.43
2nd quartile	27.04	27.29	25.43	25.93	27.25	27.49	25.47	25.87	25.86	26.55
3rd quartile	23.76	24.56	24.26	25.32	22.78	23.99	23.78	25	24.03	24.39
4th quartile	19.51	19.54	19.66	19.46	19.29	19.09	20.25	19.71	20.35	18.62
Hospital region, %										
Northeast	18.6	17	18.46	16.72	18.23	16.68	18.36	17.27	18.09	16.52
Midwest	22.36	22.14	22.22	21.97	21.92	21.69	21.83	22.09	21.48	20.6
South	39.55	38.51	39.86	39.04	40.38	39.54	40.47	39.11	40.84	40.88
West	19.48	22.36	19.46	22.26	19.47	22.08	19.33	21.53	19.6	22.01
Hospital bed size, %										
Small	21.24	19.37	22.29	20.48	22.69	21.49	23.16	21.93	23.07	22.09
Medium	29.19	28.63	28.98	28.05	28.13	27.98	27.8	27.8	28.53	28.48
Large	49.57	52	48.73	51.47	49.19	50.53	49.04	50.27	48.4	49.42
Hospital teaching/location, %										
Rural	8.98	6.45	8.77	6.36	8.76	6.37	8.6	6.1	8.56	6.76
Urban non-teaching	20.8	19.9	18.26	17.76	17.91	17.66	17.51	17.62	16.2	16.09
Urban teaching	70.22	73.66	72.97	75.88	73.33	75.97	73.89	76.28	75.24	77.15

**Table 6:** Supplementary table S3 - year-specific adjusted association of MASLD with LOS and total hospital charges, 2018–2022 Length of stay and total hospital charges were modeled using survey-weighted linear regression with log-transformed outcomes. Adjusted percent differences were derived as 100 × (exp(beta) − 1). Models adjusted for age, sex, race, primary payer, ZIP-income quartile, hospital region, hospital teaching/location status, hospital bed size, CHF, CAD, stroke, CKD, COPD, cancer, HCV infection, alcohol-related liver disease, and other liver disease MASLD: metabolic dysfunction-associated steatotic liver disease; LOS: length of stay; CI: confidence interval; CHF: congestive heart failure; CAD: coronary artery disease; CKD: chronic kidney disease; COPD: chronic obstructive pulmonary disease; HCV: hepatitis C virus

Year	LOS beta (95% CI)	P-value	Adjusted % difference in LOS	Charges beta (95% CI)	P-value	Adjusted % difference in charges
2018	0.0872 (0.0786 to 0.0957)	< 0.001	9.10%	0.1809 (0.1690 to 0.1927)	< 0.001	19.80%
2019	0.0834 (0.0756 to 0.0911)	< 0.001	8.70%	0.1823 (0.1717 to 0.1930)	< 0.001	20.00%
2020	0.0930 (0.0856 to 0.1003)	< 0.001	9.70%	0.2114 (0.2011 to 0.2218)	< 0.001	23.50%
2021	0.0902 (0.0823 to 0.0981)	< 0.001	9.40%	0.2171 (0.2067 to 0.2274)	< 0.001	24.20%
2022	0.0904 (0.0833 to 0.0974)	< 0.001	9.50%	0.1863 (0.1775 to 0.1950)	< 0.001	20.50%

Data availability

All data analyzed in this study are publicly accessible through the HCUP NIS at https://www.hcup-us.ahrq.gov.
